# Serum metabolomics modelling reveals stage-associated purine and endocannabinoid metabolic signatures in colorectal cancer progression

**DOI:** 10.3389/fonc.2026.1900337

**Published:** 2026-08-05

**Authors:** Jakub Klekowski, Paulina Fortuna, Mariusz Chabowski, Łukasz Lewandowski, Wioleta Szewczak, Karolina Mosna, Gabriela Maciejewska, Marek Zawadzki, Małgorzata Krzystek-Korpacka, Mariusz Fleszar

**Affiliations:** 1 Department of Surgery, 4th Military Clinical Hospital, Wroclaw, Poland; 2 Division of Anesthetic and Surgical Nursing, Department of Nursing, Faculty of Nursing and Midwifery, Wroclaw Medical University, Wroclaw, Poland; 3 Omics Research Center, Wroclaw Medical University, Wroclaw, Poland; 4 Department of Clinical Surgical Sciences, Faculty of Medicine, Wroclaw University of Science and Technology, Wroclaw, Poland; 5 Department of Biochemistry and Immunochemistry, Wroclaw Medical University, Wroclaw, Poland; 6 Department of Oncological Surgery, Research and Development Centre at Regional Specialist Hospital, Wroclaw, Poland

**Keywords:** colorectal cancer, endocannabinoid system, purine metabolism, serum metabolomics, tumor progression

## Abstract

**Background:**

Colorectal cancer (CRC) remains one of the major challenges in contemporary oncology. Despite advances in imaging diagnostics and histopathological assessment, we still lack tools for the early detection of micrometastases and molecular alterations. The purinergic and endocannabinoid systems play an important role in CRC pathogenesis, yet their mutual interactions are not fully understood.

**Methods:**

The study involved serum samples from 117 patients undergoing CRC surgical treatment. We performed targeted serum metabolomic profiling of purine metabolites and endocannabinoid-related lipids. Advanced statistical modeling (elastic-net regression and stepwise selection) was applied to link metabolite concentrations with TNM stage and pathological features, such as neuroinvasion and vascular infiltration.

**Results:**

AMP was the strongest marker positively correlated with higher TNM stage. On the other hand, 2-AG level showed consistent negative correlation with neuroinvasion and lymphatic nodes metastasis. Xanthine level and N stage correlation was also observed.

**Conclusions:**

The results allow us to propose a model in which AMP and 2-AG constitute opposing poles of the metabolic profile in CRC patients An increase in serum AMP was associated with more advanced CRC stage, whereas lower 2-AG levels were associated with invasive features, particularly neuroinvasion. This suggests that altered purine/nucleotide metabolism may be associated with reduced endocannabinoid tone in advanced CRC. However, the proposed interaction between purinergic signalling, COX-2 activity and 2-AG metabolism should be interpreted as a hypothesis-generating model requiring direct tissue-level validation. These findings support further investigation of purine–endocannabinoid crosstalk as a potential biologically relevant pathway in CRC progression, but therapeutic implications require direct tissue-level and functional validation.

## 1 Introduction

Colorectal cancer (CRC) is one of the most substantial challenges for global oncology and public health, being the 3^rd^ most commonly diagnosed malignancy and 2^nd^ leading cause of cancer-related deaths worldwide (1, 2). While high income countries report stable or decreasing incidence rates of CRC in population over 50 years old, there is a global concerning trend showing increasing numbers of early onset CRC diagnosed before 50 (1, 3). In Poland, CRC ranks third in terms of frequency, accounting for 11.4% of new cases in men and 9.2% in women in 2023. Mortality statistics place it second among cancer-related causes of death in men (12.8%) and third in women (11.5%). Despite the significant health burden, the epidemiological data from the past decade reveal some slight favorable downward tendency in both incidence and mortality of CRC (4, 5). Although the overall survivability rates in Poland have risen systematically over the years, reaching almost 58% for colon cancer, the numbers still remain lower than leading European countries, where survival reach nearly 70% (5).

. The standard of CRC staging includes imaging like CT or MRI and postoperative histopathological evaluation (6–8). These methods, however accurate in general evaluation, are often insufficient in detecting peritoneal metastasis, micrometastasis and molecular changes preceding tumor growth, which leads to suboptimal treatment plan (9). Moreover, commonly used tumor markers like carcinoembryonic antigen (CEA) show poor sensitivity and specificity which makes them unreliable as screening tools (9, 10). In this context the metabolomic profiling approach holds potential to fill the gap in CRC and other tumors diagnosis and staging (11, 12).

. Purinergic receptors and signaling have been extensively studied over the past two decades. These receptors fall into three groups: P0, P1, and P2, with P2 being the most important. Within the P2 group, there are two families: P2X and P2Y, each with subdivisions. The P2X receptor subunits are ATP-gated ion channels that typically induce calcium/sodium influx and potassium efflux. Seven subtypes of P2X receptors have been recognized, from P2X1 to P2X7 (13, 14). P2Y are G-protein-coupled receptors and there are eight subunits recognized, namely P2Y1, 2, 4, 6, 11-14. P2Y receptors are less selective in terms of the ligands which activate them, since they react to different di- and triphosphonucleotides such as ATP, ADP, UTP or UDP (15). ATP undergoes hydrolysis to form ADP and AMP, which can subsequently be further transformed into adenosine. Consequently, a single molecule of ATP and its hydrolytic products can activate various types of purinergic receptors. This complexity is heightened by the presence of multiple purinergic receptors on a single cell membrane (16, 17).

. The purinergic signaling system is widespread in human tissues. P2X and P2Y receptors contribute to cytokine production and inflammatory responses. For instance, P2Y2 receptors facilitate chemotaxis, granule release in neutrophils, phagocytosis in macrophages, and dendritic cell migration. P2X7 receptors are involved in releasing interleukins such as IL-1 and IL-18 by macrophages, as well as releasing prostaglandins, cathepsins, prostaglandins E2 and D2, thromboxanes, leukotrienes, and regulating autophagy and T-cell activation. Additionally, P2Y6 receptors are associated with the secretion of inflammatory cytokines like IL-8, TNF-α, and MCP-1 (18, 19).

Elevated ATP concentration in the tumor microenvironment (TME) is common due to high tumor metabolism, cells damage, hypoxia. The key characteristics of purine metabolism in TME is the balance between ATP and adenosine, maintained by ectonucleotidases CD39 and CD73. ATP in high concentration might act as a DAMP (Damage-Associated Molecular Pattern) and stimulate immunological response, however in TME it is rapidly transformed into adenosine, which exert suppressive effect on T lymphocytes and NK cells (20–22).

The endocannabinoid system (ECS) is a complex network of lipids signaling, which in the gastrointestinal tract regulates not only motility and secretion, but also inflammatory response and epithelial proliferation (23, 24). The ECS generally consists of the endocannabinoids (eCB) - which are endogenously synthesized compounds - their receptors and enzymes that break down or direct the eCB into further metabolic and signaling cascades. Two leading representatives of eCB are distinguished - arachidonoyl ethanolamine, generally called anandamide (AEA), and 2-arachidonoylglycerol (2-AG). Both are synthesized from phospholipids precursors anchored in the cell membrane and their production is triggered on demand in response to an increase of intracellular calcium level. Endocannabinoids act by biding to receptors, which belong to a family of seven-transmembrane Gi/o-coupled receptors (GPCR). There are two canonical eCB receptors: CB1 and CB2. Activation of the CB1 and CB2 receptors is said to decrease cAMP level and adenylate cyclase expression. AEA is a partial agonist of CB1 receptors (CB1R), however it has low affinity to CB2 receptors (CB2R) and it can even act as an antagonist of CB2R. On the other hand, 2-AG is an agonist of both types of the receptors. The ECS is also expanded by cannabinoid-like lipids such as oleoyl-ethanolamide (OEA) and palmitoyl-ethanolamide (PEA), which interact with various receptors. Degradation of eCBs goes in a couple of ways, but two enzymes play a leading role in the catabolism: fatty acid amide hydrolase (FAAH) and monoacylglycerol lipase (MAGL). AEA is broken down by FAAH, which results in the release of arachidonic acid (AA) ethanolamine, while 2-AG is metabolized into AA and glycerol. Notably, there are yet three immensely important alternative pathways and enzymes involved in eCB degradation. AEA and 2-AG are subjected to the activity of COX-2 (cyclooxygenase-2), LOX (lipoxygenase) and cytochrome p450, which products are significant mediators of inflammatory response (25–28).

Purinergic and endocannabinoid signaling are considered to have serious impact on CRC growth, progression and other metabolic processes. Thus far, there are only a handful of reports suggesting correlations between purinergic and endocannabinoid signaling. Jové et al. evaluated endocannabinoid-like and purine metabolites in endometrial adenocarcinoma and revealed deregulation of these metabolites, however associations between them were not studied (29). Researches on neurological diseases provide promising evidence. In P2X7R-/- mice with induced experimental autoimmune encephalomyelitis lower production of endocannabinoids compared to wild type mice was detected (30). Furthermore, Kovacs et al. reported that P2X receptors activation provokes eCB/CB1R-mediated suppression of synaptic transmission (31). In the GIT eCB and purines were associated with contractility regulation via cholinergic neurotransmission. It was proposed, that ATP by increasing acetylocholine levels enhances the production of eCBs (23). We also observe, that some pathways of downstream signaling are common for eCB and purines, like MAPK and ERK1/2 pathways as mentioned by Buzzi et al. and Tutino et al. (32–34) COX-2, which is an important element of ECS, was also associated with P2Y2 receptor according to Limami et al. (35), thus suggesting potential influence of purinergic signaling on eCB metabolism. Ca2+ is a crucial signalling ion for both systems. Activation of P2X7R is followed by an inward Ca2+ current and an increase in intracellular Ca2+ concentration (36). Similarily, stimulation of CB1R was associated with increased Ca2+ release from endoplasmic reticulum (37). Furthermore, in retinal cells activation of CB receptors primes P2X7R Ca2+ signalling (38). Reportedly, activation of P2X7R causes Ca2+-dependent release of eCBs (39). On the other hand, eCB signalling participates in ATP release, while ATP stimulation increases eCB production (37), therefore it appears that purinergic and eCB signalling create a closed circle of mutual interactions.

This study aimed to integrate targeted serum metabolomic profiles of purine metabolites and endocannabinoid-related lipids in CRC patients and to explore whether these circulating metabolites are associated with tumour stage and invasive clinicopathological features.

## 2 Material and methods

### 2.1 Studied group

The studied group was prospectively recruited among patients qualified for colorectal resections due to CRC in the 4th Military Clinical Hospital in Wroclaw, Poland. All patients participating in the study gave their written consent to be included. Anthropometric, demographic, and clinical data was were collected based on patients’ medical history. The blood samples were collected from every patient prior to the surgical procedure. The blood samples were centrifuged at 3000 rpm for 10 min in the hospital’s laboratory, and the separated serum was carefully aspirated and immediately stored at in a −80 °C freezer. After gathering the studied group, the biological material was subjected to biochemical analysis in the laboratories of the Omics Research Center of Wroclaw Medical University. The detailed characteristics of the studied group are presented in other publication (40). The final analysis included 117 subjects. In comparison with the cohort reported in the cited publication, five patients were excluded because their samples showed an unacceptably high proportion of missing matched features during MS-DIAL data processing, which did not meet the quality criteria for downstream analysis.

### 2.2 Statistical analysis

Continuous concentrations of purine metabolites, nucleosides, and endocannabinoid-related metabolites were analysed as candidate explanatory variables in relation to tumour stage and pathological characteristics. Two preprocessing layers were implemented. In the first layer, metabolites were analysed on the original concentration scale. In the second layer, metabolite concentrations were transformed using log2(x + 1). Within each layer, omics-derived variables were robustly standardised using metabolite-specific medians and interquartile ranges (IQRs). Clinical covariates were retained on their original scales.

Tumour stage was analysed primarily as an ordered categorical outcome using cumulative logit regression. TNM stage was based on the harmonised ordinal variable available in the analytical dataset and was additionally dichotomised as advanced versus non-advanced disease, defined as TNM ≥3. Additional outcomes included tumour size, nodal involvement, angioinvasion, and neuroinvasion. To reduce sparse-data instability, T category was collapsed by merging T0 with T1 and T4a/T4b into T4, yielding a four-level ordered variable. N category was collapsed by merging N1a/N1b/N1c into N1 and N2a/N2b into N2, yielding a three-level ordered variable. Angioinvasion and neuroinvasion were analysed as binary outcomes.

Two complementary exploratory modelling strategies were used. In the primary strategy, baseline omics-only models (M0) were fitted after variable selection using elastic-net penalised regression, applied separately for ordinal and binary outcomes. In the alternative strategy, bidirectional stepwise selection based on likelihood ratio tests was applied to the same outcomes and preprocessing layers, with p<0.05 used for variable entry and p≥0.10 for removal. Variables selected in M0 were subsequently refitted in unpenalised models to obtain interpretable odds ratios and confidence intervals.

For each outcome and preprocessing layer, M0 models were extended by age and sex to form adjusted models (M1). Ordinal outcomes were analysed using cumulative logit models under the proportional odds assumption, parameterised such that positive coefficients corresponded to higher outcome categories. Binary outcomes were analysed using logistic regression. The proportional odds assumption was evaluated using Brant tests for refitted ordinal models. Nested M0 and M1 models were compared using likelihood ratio tests.

Brant tests indicated potential departures from the proportional-odds assumption for selected metabolites in some ordinal models, particularly in raw-scale analyses. However, the pattern of findings remained broadly directionally consistent across preprocessing layers and complementary modelling strategies. Given the exploratory nature of the study, limited sample size, and sparse outcome strata, ordinal cumulative-logit models were retained as pragmatic descriptive models rather than strict confirmatory inferential frameworks.

Effect estimates are reported as odds ratios (ORs) with 95% confidence intervals per one IQR increase in metabolite concentration after preprocessing and robust standardisation. Analyses were performed using complete-case data for variables included in each model. Given the exploratory nature of the study, the use of data-driven variable selection, and the limited sample size relative to the number of candidate metabolites, all results should be interpreted as hypothesis-generating rather than confirmatory. Full model outputs, preprocessing details, selection paths, and diagnostic results are provided in the **Supplementary Materials**.

Because multiple metabolites, preprocessing layers, clinical outcomes, and model-selection procedures were evaluated, the probability of false-positive findings is increased. This issue is particularly relevant in the present study because data-driven variable selection was used in an exploratory setting with a limited sample size relative to the number of candidate metabolites. Therefore, selected variables should not be interpreted as stable biomarkers or as components of a validated predictive signature. Rather, the modelling results were used to identify reproducible directions of association across complementary modelling strategies and preprocessing layers. Associations that appeared only in a single model specification, depended on a single selection strategy, or showed confidence intervals overlapping the null were interpreted as less robust and were treated as hypothesis-generating observations requiring independent validation.

### 2.3 Sample preparation and targeted metabolomic analysis

#### 2.3.1 Purines

Purine metabolites were quantified using a targeted UPLC-MS/MS method. Prior to extraction, serum samples were diluted four-fold with ultrapure water. Subsequently, 50 μL of diluted serum was mixed with 500 μL of 80% methanol containing 5 mM ammonium formate to precipitate proteins and extract purine metabolites. Samples were vortex-mixed at 1100 rpm for 10 min and centrifuged for 7 min. Following centrifugation, 450 μL of the supernatant was transferred to a clean tube and evaporated to dryness using a SpeedVac concentrator for 1.5 h. The dried extracts were reconstituted in 80 μL of water, vortex-mixed, and transferred to autosampler vials for LC-MS/MS analysis.

Chromatographic separation was performed using an ACQUITY UPLC I-Class system (Waters, Milford, MA, USA) equipped with an Atlantis™ Premier BEH C18 AX column (1.7 μm particle size). The column temperature was maintained at 40 °C, while the autosampler temperature was set at 10 °C. Mobile phase A consisted of water containing 0.1% formic acid, and mobile phase B consisted of acetonitrile containing 0.1% formic acid. The flow rate was 0.25 mL/min. The gradient program was as follows: 0–2.0 min, 2% B; 2.0–6.0 min, linear increase to 35% B; 6.0–7.6 min, linear increase to 95% B; 7.6–8.6 min, held at 95% B; 8.6–9.0 min, returned to 2% B, followed by re-equilibration until 11.0 min.

Mass spectrometric detection was carried out on a Xevo TQ Absolute triple quadrupole mass spectrometer (Waters Corporation) equipped with an electrospray ionization source operating in positive ionization mode. Source parameters were set as follows: capillary voltage, 3.0 kV; source temperature, 150 °C; desolvation temperature, 350 °C; cone gas flow, 150 L/h; and desolvation gas flow, 650 L/h. Data were acquired in multiple reaction monitoring (MRM) mode. Quantification was based on the following transitions (precursor → product ion, m/z): adenine 136.07→119.07, hypoxanthine 137.00→110.05, xanthine 153.06→110.05, uric acid 169.00→140.97, guanine 152.03→110.05, adenosine 267.98→136.00, inosine 268.99→137.04, guanosine 283.99→152.01, xanthosine 285.05→152.94, 2-deoxyadenosine 252.00→136.01, 2-deoxyguanosine 267.99→151.99, 2-deoxyinosine 252.98→206.96, AMP 347.91→136.02, and GMP 363.99→322.90.

Calibration curves were prepared using ten concentration levels for each analyte. Depending on the compound, calibration ranges were as follows: 0.063–32 nM for 2′-deoxyadenosine, 0.125–64 nM for 2′-deoxyguanosine, 2′-deoxyinosine and adenine hemisulfate, 0.977–500 nM for AMP, adenosine, guanosine, xanthosine, adenine and guanine, 15.625–4000 nM for inosine and hypoxanthine, 15.625–8000 nM for xanthine, and 78.125–40000 nM for uric acid. Quantitative data processing was performed using Waters Connect software 3.2 (Waters, Milford, MA, USA).

#### 2.3.2 Endocannabinoid

Palmitoylethanolamide (PEA), oleoylethanolamide (OEA), anandamide (AEA), 2-arachidonoylglycerol (2-AG), 1-arachidonoylglycerol (1-AG), stearoylethanolamide (SEA), and their isotope-labelled internal standards (PEA-d4, OEA-d2, AEA-d8, 2-AG-d5, 1-AG-d5, and SEA-d3) were purchased from Cayman Chemical (Ann Arbor, MI, USA). Methanol, acetonitrile (ACN), toluene, water, and formic acid (FA) were obtained from Merck Millipore (Warsaw, Poland).

##### 2.3.2.1 Sample preparation

Endocannabinoids were extracted according to a previously validated liquid–liquid protocol (41). Briefly, 100 μL of serum sample or calibration standard was transferred into a 2.0 mL polypropylene tube and mixed with 20 μL of internal standard solution prepared in methanol containing 160 nM PEA-d4, 40 nM OEA-d2 and AEA-d8, and 400 nM 2-AG-d5 and 1-AG-d5. After vortex-mixing for 1 min, samples were extracted with 400 μL of toluene and mixed for 5 min at room temperature. Samples were subsequently centrifuged at 12, 500 rpm for 5 min at 4 °C. An aliquot of 350 μL of the organic phase was collected and evaporated to dryness under a gentle stream of nitrogen. The residue was reconstituted in 120 μL of acetonitrile/water/formic acid (25:75:0.1, v/v/v), vortex-mixed, and transferred into glass autosampler vials for LC–MS/MS analysis.

##### 2.3.2.2 UHPLC–MS/MS analysis

Chromatographic separation was performed using an ACQUITY UPLC BEH C18 column (100 × 1.0 mm, 1.7 μm; Waters, Milford, MA, USA) maintained at 60 °C. Analyses were carried out on an ACQUITY UPLC system coupled to a Xevo Absolute Triple Quadrupole mass spectrometer (Waters, Milford, MA, USA) equipped with an electrospray ionization (ESI) source operating in positive ion mode.

The mobile phases consisted of water containing 0.1% formic acid (solvent A) and acetonitrile containing 0.1% formic acid (solvent B). The flow rate was 0.18 mL/min and the injection volume was 12 μL. The gradient program was as follows: 50% B from 0 to 1.5 min, increased to 80% B at 8.0 min, then to 100% B at 9.0 min, maintained until 14.0 min, followed by re-equilibration to initial conditions until 18.0 min.

Mass spectrometric detection was performed in multiple reaction monitoring (MRM) mode. The source parameters were set as follows: capillary voltage 2.5 kV, source temperature 150 °C, desolvation temperature 400 °C, cone gas flow 150 L/h, desolvation gas flow 800 L/h, and collision gas flow 0.15 mL/min. Quantification was performed using stable isotope dilution and calibration curves generated from authentic standards. Data acquisition and processing were carried out using MassLynx Software version 4.2 (Waters, Milford, MA, USA).

## 3 Results

### 3.1 Modelling the primary outcomes

In ordinal models of TNM stage analysed on the raw concentration scale, AMP showed the most consistent positive association with higher TNM categories across both modelling strategies. The magnitude and direction of the association remained materially unchanged after adjustment for age and sex. In contrast, adenosine demonstrated a recurrent inverse association in the stepwise models only. Xanthine showed a weaker positive pattern restricted to the penalised approach and attenuated after adjustment. AEA and 2-deoxyinosine showed inverse directions in penalised models, although without robust statistical support. In complementary binary analyses contrasting advanced versus non-advanced disease (TNM ≥3), AMP was retained only in the stepwise models, where it showed a persistent positive association after adjustment. Overall, the raw-scale analyses identified AMP as the most reproducible metabolite associated with higher TNM stage, whereas the remaining associations were weaker and more strategy-dependent.

In analyses performed after log2 transformation, the association between AMP and higher TNM stage remained consistent across both ordinal and binary formulations of the outcome. In addition, 2-deoxyadenosine demonstrated a reproducible positive association with higher ordinal TNM categories across both modelling strategies, with little attenuation after adjustment for age and sex. Hypoxanthine showed positive associations in the binary TNM ≥3 analyses within the stepwise framework and weaker ordinal signals in the penalised models. Adenine demonstrated an inverse direction in penalised ordinal models, although without statistical support. Compared with the raw-scale analyses, the log2-transformed analyses showed greater consistency for selected purine-related metabolites, particularly AMP and 2-deoxyadenosine, whereas endocannabinoid-related signals were less prominent.

**Figure 1:**
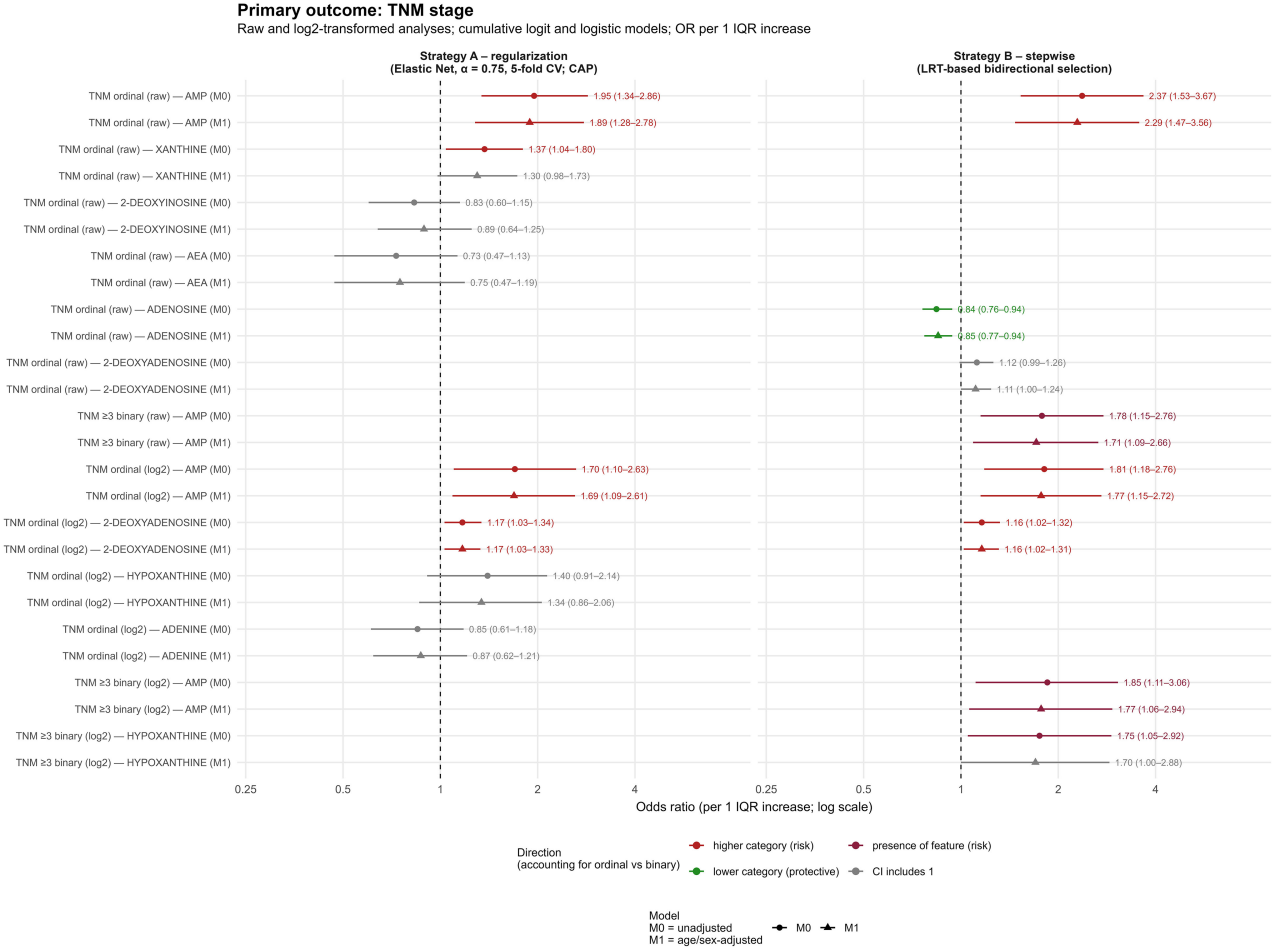
Modelling the primary outcome — visualization.

The results are shown in **Table 1**, **Table 2**, and **Figure 1**.

**Table 1:** Modelling the primary outcome — TNM (raw-scale analysis).

Modelled	Metabolite	Strategy A: unadjusted (M0)	Strategy A: adjusted (M1)	Strategy B: unadjusted (M0)	Strategy B: adjusted (M1)
TNM (ordinal)	AMP	1.95 (1.34–2.86), p<0.001	1.89 (1.28–2.78), p=0.001	2.37 (1.53–3.67), p<0.001	2.29 (1.47–3.56), p<0.001
TNM (ordinal)	XANTHINE	1.37 (1.04–1.80), p=0.025	1.30 (0.98–1.73), p=0.067	—	—
TNM (ordinal)	2-DEOXYINOSINE	0.83 (0.60–1.15), p=0.267	0.89 (0.64–1.25), p=0.503	—	—
TNM (ordinal)	AEA	0.73 (0.47–1.13), p=0.153	0.75 (0.47–1.19), p=0.223	—	—
TNM (ordinal)	ADENOSINE	—	—	0.84 (0.76–0.94), p=0.001	0.85 (0.77–0.94), p=0.002
TNM (ordinal)	2-DEOXYADENOSINE	—	—	1.12 (0.99–1.26), p=0.073	1.11 (1.00–1.24), p=0.054
TNM ≥3 (binomial)	AMP	—	—	1.78 (1.15–2.76), p=0.010	1.71 (1.09–2.66), p=0.019

OR expressed per 1 IQR increase in metabolite concentration following preprocessing and robust scaling. Raw analyses were performed on original concentration values. Strategy A: elastic-net variable selection followed by unpenalised refitting. Strategy B: bidirectional stepwise selection based on likelihood ratio tests. M1 models adjusted for age and sex. More detailed model outputs are provided in the **Supplementary Materials**.

**Table 2:** Modelling the primary outcome — TNM (log2-transformed analysis).

Modelled	Metabolite	Strategy A: unadjusted (M0)	Strategy A: adjusted (M1)	Strategy B: unadjusted (M0)	Strategy B: adjusted (M1)
TNM (ordinal)	AMP	1.70 (1.10–2.63), p=0.016	1.69 (1.09–2.61), p=0.019	1.81 (1.18–2.76), p=0.007	1.77 (1.15–2.72), p=0.009
TNM (ordinal)	2-DEOXYADENOSINE	1.17 (1.03–1.34), p=0.017	1.17 (1.03–1.33), p=0.016	1.16 (1.02–1.32), p=0.028	1.16 (1.02–1.31), p=0.023
TNM (ordinal)	HYPOXANTHINE	1.40 (0.91–2.14), p=0.122	1.34 (0.86–2.06), p=0.192	—	—
TNM (ordinal)	ADENINE	0.85 (0.61–1.18), p=0.326	0.87 (0.62–1.21), p=0.400	—	—
TNM ≥3 (binomial)	AMP	—	—	1.85 (1.11–3.06), p=0.017	1.77 (1.06–2.94), p=0.029
TNM ≥3 (binomial)	HYPOXANTHINE	—	—	1.75 (1.05–2.92), p=0.032	1.70 (1.00–2.88), p=0.050

OR expressed per 1 IQR increase in metabolite concentration following preprocessing and robust scaling. Log2 analyses were performed after log2(x + 1) transformation. Strategy A: elastic-net variable selection followed by unpenalised refitting. Strategy B: bidirectional stepwise selection based on likelihood ratio tests. M1 models adjusted for age and sex. More detailed model outputs are provided in the **Supplementary Materials**.

### 3.2 Modelling the secondary outcomes

In analyses of tumour size (T category) performed on the raw concentration scale, no strong or reproducible metabolite associations were identified across modelling strategies. Adenosine showed a weak inverse association with higher T categories in the penalised models, reaching borderline statistical support, whereas PEA demonstrated a similar inverse direction without robust significance. Guanine showed a weak positive direction, and 2-deoxyadenosine demonstrated minimal association with tumour size. In contrast, the stepwise models did not retain any metabolite for T category, indicating limited reproducibility of these findings.

In ordinal models of nodal involvement (N category), xanthine demonstrated the most consistent positive association with higher nodal categories across both modelling strategies, with only modest attenuation after adjustment for age and sex. AMP showed a positive association in the stepwise models, whereas the corresponding penalised estimates were weaker and statistically uncertain. Guanine and 2-deoxyadenosine showed weaker positive patterns in penalised models without clear support after adjustment. Overall, the raw-scale analyses suggested a more reproducible signal for nodal involvement than for tumour size, particularly for xanthine.

In binary analyses of angioinvasion, no robust metabolite associations were identified. A weak inverse pattern for 2AG was observed in the stepwise models, although without clear statistical support after adjustment.

In analyses of neuroinvasion performed on the raw concentration scale, 2AG showed a recurrent inverse direction across both modelling strategies, although confidence intervals included the null after adjustment. 2-deoxyadenosine demonstrated weak positive estimates in the penalised models without robust statistical support. Overall, the raw-scale neuroinvasion analyses suggested a possible inverse association for 2AG, although the findings remained exploratory and relatively unstable across modelling frameworks.

In analyses performed after log2 transformation, the pattern for tumour size remained generally weak and inconsistent across modelling approaches. However, PEA demonstrated a stronger inverse association in the adjusted penalised models, whereas inosine showed a similar inverse trend without clear statistical support. No metabolite was retained in the stepwise models for T category.

For nodal involvement, the log2-transformed analyses showed a more coherent pattern across several metabolites. Xanthine remained positively associated with higher nodal categories in the penalised models after adjustment, while AMP also demonstrated a persistent positive association. In contrast, 2AG showed a recurrent inverse association with nodal involvement in the penalised framework. Hypoxanthine emerged as an additional positive signal in the stepwise models. Together, these findings suggested a mixed pattern involving both purine-related metabolites and endocannabinoid-related pathways in relation to nodal disease burden.

In analyses of angioinvasion after log2 transformation, 2AG demonstrated an inverse association in the adjusted stepwise models, although the effect size remained moderate and was not consistently reproduced across modelling strategies.

The most consistent findings across the secondary outcomes were observed for neuroinvasion. In both penalised and stepwise models, 2AG showed a reproducible inverse association that remained materially unchanged after adjustment for age and sex. In contrast, 2-deoxyadenosine demonstrated weak positive estimates without robust statistical support. Compared with the raw-scale analyses, the log2-transformed models strengthened the consistency of the inverse 2AG signal for neuroinvasion.

**Figure 2:**
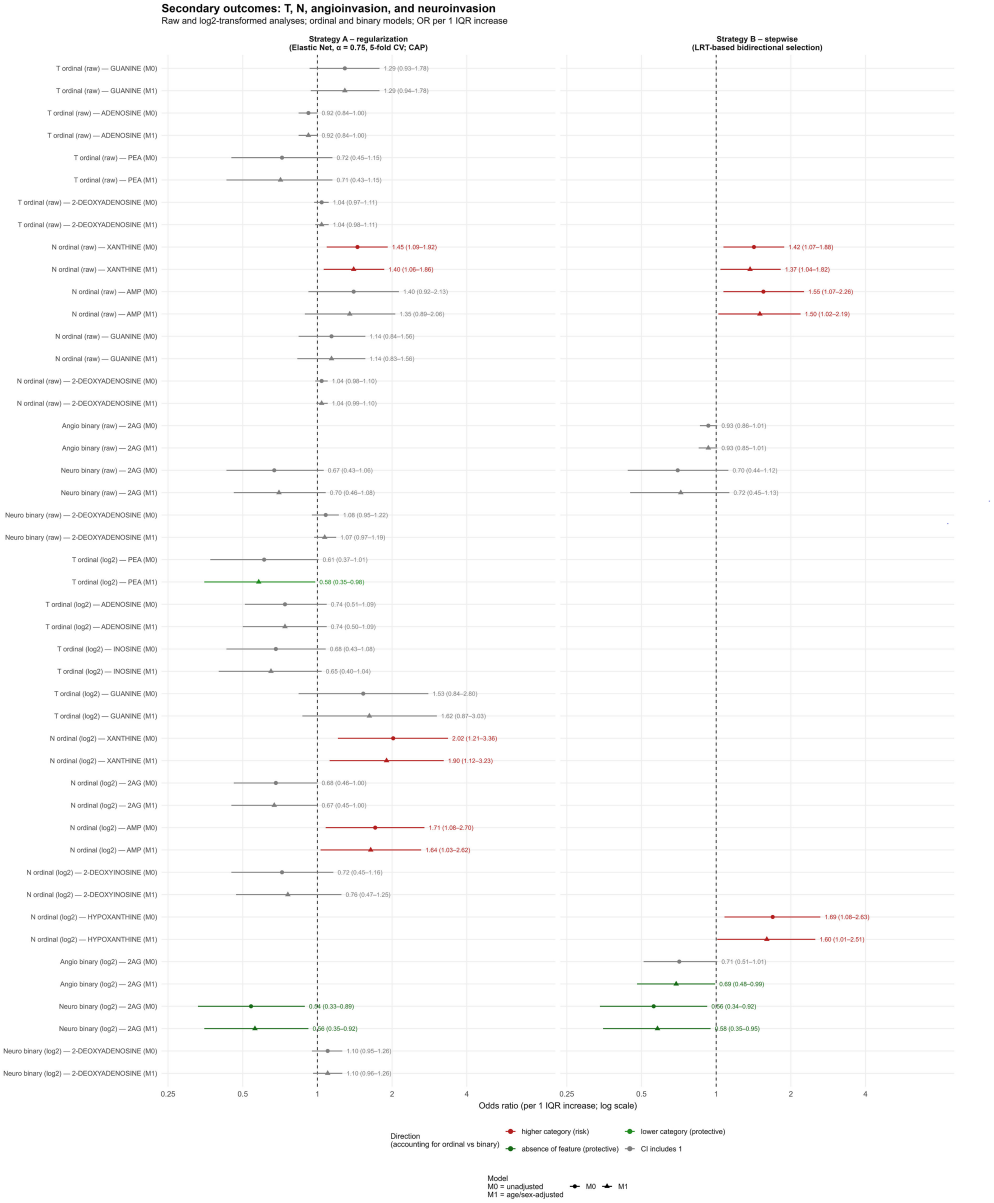
Modelling the secondary outcomes — visualization.

The results are shown in **Table 3**, **Table 4**, and **Figure 2**.

**Table 3:** Modelling the secondary outcomes — raw-scale analysis.

Modelled	Metabolite	Strategy A: unadjusted (M0)	Strategy A: adjusted (M1)	Strategy B: unadjusted (M0)	Strategy B: adjusted (M1)
T (ordinal)	GUANINE	1.29 (0.93–1.78), p=0.122	1.29 (0.94–1.78), p=0.121	—	—
T (ordinal)	ADENOSINE	0.92 (0.84–1.00), p=0.049	0.92 (0.84–1.00), p=0.051	—	—
T (ordinal)	PEA	0.72 (0.45–1.15), p=0.165	0.71 (0.43–1.15), p=0.160	—	—
T (ordinal)	2-DEOXYADENOSINE	1.04 (0.97–1.11), p=0.250	1.04 (0.98–1.11), p=0.238	—	—
N (ordinal)	XANTHINE	1.45 (1.09–1.92), p=0.010	1.40 (1.06–1.86), p=0.018	1.42 (1.07–1.88), p=0.014	1.37 (1.04–1.82), p=0.026
N (ordinal)	AMP	1.40 (0.92–2.13), p=0.112	1.35 (0.89–2.06), p=0.162	1.55 (1.07–2.26), p=0.022	1.50 (1.02–2.19), p=0.037
N (ordinal)	GUANINE	1.14 (0.84–1.56), p=0.405	1.14 (0.83–1.56), p=0.410	—	—
N (ordinal)	2-DEOXYADENOSINE	1.04 (0.98–1.10), p=0.175	1.04 (0.99–1.10), p=0.145	—	—
Angioinvasion (binomial)	2AG	—	—	0.93 (0.86–1.01), p=0.107	0.93 (0.85–1.01), p=0.101
Neuroinvasion (binomial)	2AG	0.67 (0.43–1.06), p=0.086	0.70 (0.46–1.08), p=0.107	0.70 (0.44–1.12), p=0.135	0.72 (0.45–1.13), p=0.154
Neuroinvasion (binomial)	2-DEOXYADENOSINE	1.08 (0.95–1.22), p=0.231	1.07 (0.97–1.19), p=0.180	—	—

OR expressed per 1 IQR increase in metabolite concentration following preprocessing and robust scaling. Raw analyses were performed on original concentration values. Strategy A: elastic-net variable selection followed by unpenalised refitting. Strategy B: bidirectional stepwise selection based on likelihood ratio tests. M1 models adjusted for age and sex. More detailed model outputs are provided in the **Supplementary Materials**.

**Table 4:** Modelling the secondary outcomes — log2-transformed analysis.

Modelled	Metabolite	Strategy A: unadjusted (M0)	Strategy A: adjusted (M1)	Strategy B: unadjusted (M0)	Strategy B: adjusted (M1)
T (ordinal)	PEA	0.61 (0.37–1.01), p=0.054	0.58 (0.35–0.98), p=0.043	—	—
T (ordinal)	ADENOSINE	0.74 (0.51–1.09), p=0.129	0.74 (0.50–1.09), p=0.123	—	—
T (ordinal)	INOSINE	0.68 (0.43–1.08), p=0.102	0.65 (0.40–1.04), p=0.075	—	—
T (ordinal)	GUANINE	1.53 (0.84–2.80), p=0.168	1.62 (0.87–3.03), p=0.131	—	—
N (ordinal)	XANTHINE	2.02 (1.21–3.36), p=0.007	1.90 (1.12–3.23), p=0.017	—	—
N (ordinal)	2AG	0.68 (0.46–1.00), p=0.048	0.67 (0.45–1.00), p=0.049	—	—
N (ordinal)	AMP	1.71 (1.08–2.70), p=0.022	1.64 (1.03–2.62), p=0.037	—	—
N (ordinal)	2-DEOXYINOSINE	0.72 (0.45–1.16), p=0.181	0.76 (0.47–1.25), p=0.281	—	—
N (ordinal)	HYPOXANTHINE	—	—	1.69 (1.08–2.63), p=0.021	1.60 (1.01–2.51), p=0.043
Angioinvasion (binomial)	2AG	—	—	0.71 (0.51–1.01), p=0.056	0.69 (0.48–0.99), p=0.046
Neuroinvasion (binomial)	2AG	0.54 (0.33–0.89), p=0.015	0.56 (0.35–0.92), p=0.022	0.56 (0.34–0.92), p=0.021	0.58 (0.35–0.95), p=0.031
Neuroinvasion (binomial)	2-DEOXYADENOSINE	1.10 (0.95–1.26), p=0.199	1.10 (0.96–1.26), p=0.170	—	—

OR expressed per 1 IQR increase in metabolite concentration following preprocessing and robust scaling. Log2 analyses were performed after log2(x + 1) transformation. Strategy A: elastic-net variable selection followed by unpenalised refitting. Strategy B: bidirectional stepwise selection based on likelihood ratio tests. M1 models adjusted for age and sex. More detailed model outputs are provided in the **Supplementary Materials**.

## 4 Discussion

The results of our study provide evidence that serum profiles of purinergic and endocannabinoid metabolites could be considered as biomarkers of CRC aggressiveness. In the context of oncogenesis, the positive correlation of AMP levels with pathological advancement of the tumor and the negative correlation of 2-AG levels with neuroinvasion shed a new light on pathological coupling of these systems.

In this study, AMP concentration increased consistently with higher TNM stage, suggesting that this metabolite may serve as a circulating marker of altered purine/nucleotide metabolism associated with disease advancement. In the tumor’s microenvironment ATP is being released in high amounts then it is forwarded to CD39/CD73 axis and decomposed into AMP (42–44). In previous studies on CRC and other neoplasms it has been established that accumulation of ATP-degradation products and activation of P2X7 favors tumor growth and is a sign of wore prognosis (21, 36, 45). Therefore, higher serum AMP levels in patients with advanced CRC may reflect systemic purine metabolic remodelling accompanying tumour progression, although the present serum-based data do not establish its tumour-specific origin.

An important finding of our research is the link of low 2-AG level with neuroinvasion. The correlation with angioinvasion, due to its weak statistical relationship, requires further studies. The pre-clinical evidence indicate that 2-AG as a complete agonist of CB1 and CB2 is a suppressor of cancer cells migration. The decrease of this eCB could be attributed to overexpression of MAGL in tumor cells, which not only degrades protective 2-AG, but also provides substrates for eicosanoid synthesis. In CRC cell lines inhibiting MAGL caused reduced proliferation and increased apoptosis through modulation of Bcl-2 and Bax expression, promoted cells sensitivity to 5-fluorouracil, inhibited cells migration and modified epithelial to mesenchymal transition (EMT) markers by increasing E-cadherin and reducing vimentin and SNAI1 (24, 28, 46). Based on these mechanisms, our findings suggest that lower serum 2-AG may be compatible with reduced endocannabinoid tone in patients with more invasive tumour features; however, this interpretation requires direct validation in CRC tissue and functional models.

When comparing our results with the existing literature there is a mechanistic discrepancy between our findings and the literature in the purines-ECS interaction. Neurobiological studies show, that in the homeostasis high concentration of purines and activation of P2X7 should stimulate on-demand synthesis of 2-AG through influx of Ca^2+^. Furthermore, the sustained increase in Ca^2+^ is expected to not only activate DAGL to produce 2-AG, but also to inhibit MAGL responsible for 2-AG hydrolysis (47–49).

The inverse relationship between serum AMP and 2-AG should be interpreted as a hypothesis-generating observation rather than direct evidence of a defined regulatory pathway. Based on available pharmacological data, P2Y2 receptors are activated primarily by ATP and UTP, whereas AMP is not considered an active P2Y receptor ligand (50). Therefore, elevated serum AMP in advanced CRC should not be described as a direct activator of P2Y2. Rather, it may represent a circulating marker of altered purine/nucleotide metabolism associated with tumour progression. Literature indicates that P2Y2 activation can engage Gq/PLC/IP3/DAG/Ca²^+^ signaling and contribute to inflammatory mediator release, including arachidonic acid, prostaglandins and nitric oxide (51). In intestinal inflammatory models, P2Y2 signaling has also been linked to COX-2-dependent PGE2 production (51, 52). In parallel, 2-AG is a biologically active endocannabinoid acting through cannabinoid receptors and is mainly inactivated by MAGL and α/β-hydrolase domain-containing protein 6 (ABHD6) (48). Signorello and Leoncini further demonstrated that 2-AG can activate CB1-dependent calcium/calmodulin-dependent protein kinase kinase β/AMP-activated protein kinase α subunit (CaMKKβ/AMPKα) signaling in human platelets (53). Experimental data further show that ATP-dependent purinergic signaling can increase 2-AG production via Ca²^+^/PLC/DAGLβ-dependent mechanisms, with a potential minor contribution of P2Y2/4 signaling (48). Thus, our finding of reduced 2-AG in more invasive disease does not prove direct P2Y2/COX-2-mediated degradation of 2-AG, but may indicate a broader imbalance between purine-associated inflammatory signaling and endocannabinoid tone. Direct assessment of P2Y2, COX-2, MAGL, CB1 and CB2 expression or activity in CRC tissue will be necessary to validate this proposed mechanism.

From a translational perspective, the present serum findings support the view that purine and endocannabinoid alterations in CRC are not merely secondary metabolic by-products, but may reflect clinically relevant vulnerabilities within the tumour–host system. This interpretation is consistent with recent multi-omic evidence showing that perturbation of purine metabolism can define targetable dependencies in KRAS-mutant tumours: in patient-derived CRC models, trametinib induced dysregulation of purine biosynthesis, including reduced guanine levels and suppression of the purine biosynthetic enzyme GART, which created sensitivity to the purine analogue thioguanine in combination with MEK inhibition (54). In parallel, integrative transcriptomic, immunohistochemical and single-cell analyses have shown that purine metabolism-related genes and proteins, including ADSL, APRT, ADCY3, NME3 and NME6, are associated with CRC prognosis and clinicopathological progression, further supporting the clinical relevance of purine pathway remodelling (55). These observations strengthen the interpretation of our AMP- and hypoxanthine-related associations with tumour advancement as markers of broader purine metabolic rewiring rather than isolated circulating changes. In addition, the inverse association between 2-AG and neuroinvasion may have immunological implications. Experimental CRC data indicate that CB1 activation suppresses tumour cell proliferation, migration and invasion, and reduces M2 macrophage polarization through EGFR downregulation; conversely, CB1 inhibition enhances tumour growth-related phenotypes and M2 polarization (56). Thus, reduced circulating 2-AG in more invasive disease may indicate loss of endogenous cannabinoid tone, potentially weakening CB1/CB2-dependent restraint of tumour invasion and tumour-promoting macrophage polarization. Taken together, these data suggest that the serum purine–endocannabinoid pattern observed in this study may reflect both metabolic progression and altered tumour–immune communication, although direct tissue-level validation of receptor expression, ectonucleotidase activity and macrophage polarization will be required.

## 5 Conclusions

The identified correlations allow us to propose that AMP and 2-AG may represent opposing components of the circulating metabolic profile associated with CRC progression. Increased serum AMP may reflect altered purine/nucleotide metabolism in advanced disease, whereas reduced serum 2-AG may indicate impaired endocannabinoid tone associated with invasive tumour features. However, the proposed interaction between purinergic signalling, COX-2-related inflammatory metabolism and 2-AG requires direct validation in CRC tissue, including assessment of P2Y2, COX-2, MAGL, CB1 and CB2 expression or activity. Future studies should further explore the relationships between the endocannabinoid and purinergic systems in the tumour microenvironment.

A limitation of the present study is that metabolite concentrations were measured in serum; therefore, the tumour-specific origin of the observed AMP and 2-AG alterations cannot be established. Although these circulating changes were associated with CRC stage and invasive clinicopathological features, they should not be interpreted as direct evidence of metabolic activation within the tumour microenvironment. Paired tumour and adjacent non-tumour tissue analyses, together with validation of purinergic and endocannabinoid pathway components at the tissue level, will be required to confirm whether the serum alterations reflect intratumoral metabolic reprogramming. In addition, the study did not include a healthy control group. Therefore, the present findings should be interpreted as stage-associated differences within CRC rather than CRC-specific diagnostic alterations. Future studies including healthy controls and benign colorectal disease controls are needed to determine the disease specificity of these metabolic patterns. A further limitation is the increased risk of false-positive findings arising from the combination of multiple tested metabolites, several clinical endpoints, two preprocessing layers, and data-driven model-selection procedures. Although we compared directions of association across complementary modelling strategies and preprocessing layers, no formal external validation or resampling-based stability analysis was performed. Consequently, the selected associations should be regarded as prioritised exploratory signals rather than validated biomarkers. Moreover, an additional limitation concerns residual confounding and the interpretation of adjusted models. The adjusted analyses included age and sex and should therefore be interpreted as minimally adjusted rather than fully confounder-controlled models. Other factors, including BMI, metabolic comorbidities, inflammatory status, medication exposure, nutritional changes and perioperative clinical status, may influence circulating purine and endocannabinoid-related metabolites. However, some of these variables may also be closely related to CRC progression itself and may represent downstream consequences or biological correlates of more advanced disease rather than purely baseline confounders. Therefore, broad *post hoc* adjustment without a prespecified causal framework could introduce overadjustment or collider-related bias and might attenuate disease-related metabolic signals. In addition, because several outcomes were ordinal and unevenly distributed across categories, expanding the adjustment set would have reduced the effective events-per-parameter ratio and increased the risk of unstable estimates and sparse-data bias. Future studies should include larger cohorts with richer clinical phenotyping and prespecified causal models to separate baseline confounding from inflammatory, treatment-related and metabolic features that may lie on or near the pathway between CRC progression and circulating metabolite alterations.

## Data Availability

The raw data supporting the conclusions of this article will be made available by the authors, without undue reservation.
