# Supplementary material for: Serum metabolomics modelling reveals stage-associated purine and endocannabinoid metabolic signatures in colorectal cancer progression
Source: Front Oncol. 2026 Aug 5;16:1900337. doi: 10.3389/fonc.2026.1900337 (PMC13485730; doi:10.3389/fonc.2026.1900337)
Supplement: Supplementary file 1 [file DataSheet1.pdf]

README – Supplementary Model Outputs

Project overview

This supplementary package contains detailed modelling outputs for exploratory analyses of purine metabolites, nucleosides, and endocannabinoid-related metabolites in relation to tumour stage and pathological characteristics.

The analyses were performed using two complementary modelling frameworks:

1. Penalised variable selection (elastic-net) followed by unpenalised model refitting.
2. Bidirectional stepwise likelihood-ratio-test-based variable selection followed by unpenalised model refitting.

Two preprocessing layers were analysed separately:

- raw concentration scale,
- log2(x + 1)-transformed concentration scale.

All omics-derived variables were robustly standardised using metabolite-specific medians and interquartile ranges (IQRs).

The outputs are distributed across two Excel workbooks:

- `suppl_output_penalized.xlsx`
- `suppl_output_stepwise.xlsx`

File 1

`suppl_output_penalized.xlsx`

This workbook contains outputs generated using the penalised-selection workflow (elastic-net variable selection followed by unpenalised refitting).

General structure

The workbook is organised into separate worksheets corresponding to:

- preprocessing layers,
- analysed outcomes,
- model types.

The suffix:

- `_raw` refers to analyses performed on original metabolite concentrations,
- `_log2` refers to analyses performed after log2(x + 1) transformation.

Scaling sheets

`SCALING_raw`

`SCALING_log2`

These sheets contain preprocessing information for metabolite scaling.
For each metabolite, the following information is provided:

- median,
- interquartile range (IQR),
- number of finite observations.

These values were used for robust standardisation.
The sheets also contain metadata describing:

- preprocessing layer,
- transformation type,
- modelling directionality settings.

Primary outcome sheets

`TNM_ORD_raw`

`TNM_ORD_log2`

Ordinal cumulative-logit models for harmonised TNM stage.
The sheets contain:

- variables selected during penalised modelling,
- refitted unpenalised model coefficients,
- odds ratios and confidence intervals,
- likelihood-ratio-test comparisons between M0 and M1 models,
- Brant proportional-odds diagnostics.

M0 models include omics-derived variables only.
M1 models additionally include:

- age,
- sex.

`TNM_BIN_raw`

`TNM_BIN_log2`

Binary logistic-regression analyses comparing:

- TNM ≥ 3
- versus
- TNM < 3.

The sheets contain:

- selected metabolites,
- unpenalised refitted coefficients,
- odds ratios,
- confidence intervals,
- likelihood-ratio-test comparisons.

Secondary outcome sheets

`T_ORD_raw`

`T_ORD_log2`

Ordinal cumulative-logit models for tumour size category.
T categories were collapsed as follows:

- T0 merged with T1,
- T4a and T4b merged into T4.

`N_ORD_raw`

`N_ORD_log2`

Ordinal cumulative-logit models for nodal involvement.
N categories were collapsed as follows:

- N1a/N1b/N1c merged into N1,
- N2a/N2b merged into N2.

`ANGIO_BIN_raw`

`ANGIO_BIN_log2`

Binary logistic-regression models for angioinvasion.

`NEURO_BIN_raw`

`NEURO_BIN_log2`

Binary logistic-regression models for neuroinvasion.

Structure within modelling sheets

Most modelling worksheets contain the following sections:

Selected omics variables (M0)

Variables retained after penalised selection.

Model M0 coefficients

Refitted unpenalised coefficients for omics-only models.
Reported statistics include:

- regression coefficients,
- standard errors,
- z statistics,
- p-values,
- odds ratios (OR),
- 95% confidence intervals.

Model M1 coefficients (+age+sex)

Refitted unpenalised coefficients after adjustment for:

- age,
- sex.

LRT M0 vs M1

Likelihood-ratio-test comparison between:

- M0 (omics-only),
- M1 (omics + age + sex).

Brant test (M0 / M1)

Assessment of the proportional-odds assumption for ordinal models.

These sections are present only for ordinal outcomes.

File 2

suppl_output_stepwise.xlsx

This workbook contains outputs generated using bidirectional stepwise variable selection based on likelihood-ratio tests.

The overall workbook structure mirrors the penalised-analysis workbook.

The preprocessing layers, outcomes, and worksheet naming conventions are identical.

Stepwise selection procedure

Variable selection was performed using iterative bidirectional likelihood-ratio-test-based selection.

Selection thresholds:

- variable entry: $p < 0.05$,
- variable removal: $p \geq 0.10$.

Both ordinal cumulative-logit models and binary logistic-regression models were analysed.

Selected variables were subsequently refitted using unpenalised regression models.

Additional sections specific to the stepwise workbook

Stepwise history (M0)

These sections document the sequence of variable-selection steps.

The following information is provided:

- step number,
- action performed (ADD or DROP),
- metabolite name,
- likelihood-ratio-test p-value.

This allows reconstruction of the model-building pathway.

Interpretation notes

All analyses should be interpreted as exploratory and hypothesis-generating.

The study involved:

- multiple correlated metabolites,
- relatively limited sample size,
- data-driven variable-selection procedures.

Consequently, the reported associations should not be interpreted as confirmatory evidence of causal or independent biological effects.

The outputs are provided to maximise analytical transparency and reproducibility.

Software environment

Analyses were performed in R.

Main packages used included:

- VGAM,
- glmnet,
- ordinalNet,
- MASS,
- brant,
- openxlsx,
- dplyr.

Ordinal outcomes were analysed using cumulative-logit models under the proportional-odds assumption.

Binary outcomes were analysed using logistic regression.
